# Determinants of off-label drug acceptance in pregnancy: insights from an extended UTAUT model

**DOI:** 10.3389/fphar.2026.1850058

**Published:** 2026-09-21

**Authors:** Chunhong Liang, Fayan Que, Pingzhi Peng, Lijuan Zhou, Qian Qiu, Yi Pang, Xueyan Zhang

**Affiliations:** 1 Office of Drug Clinical Trial Institution, The First People’s Hospital of Yulin, The Sixth Affiliated Hospital of Guangxi Medical University, Yulin, Guangxi, China; 2 Department of Obstetrics and Gynecology, The First People’s Hospital of Yulin, The Sixth Affiliated Hospital of Guangxi Medical University, Yulin, Guangxi, China; 3 Department of Pharmacy, The First People’s Hospital of Yulin, The Sixth Affiliated Hospital of Guangxi Medical University, Yulin, Guangxi, China; 4 Center for Reproductive Medicine, The First People’s Hospital of Yulin, The Sixth Affiliated Hospital of Guangxi Medical University, Yulin, Guangxi, China

**Keywords:** cross-sectional survey, informed consent, off-label drug use, pregnant women, trust, UTAUT model

## Abstract

**Background:**

Off-label drug use (OLDU) is prevalent in obstetrics due to the scarcity of medications approved for pregnancy, raising potential risks and ethical challenges, particularly concerning informed consent. In this study, we aimed to identify the key determinants of OLDU acceptance among pregnant women using an extended Unified Theory of Acceptance and Use of Technology (UTAUT) model.

**Methods:**

A cross-sectional survey of 506 pregnant women was conducted at a tertiary hospital using a validated UTAUT-based questionnaire. Multivariable linear regression identified factors associated with behavioral intention. Six perceived risk-by-subgroup interactions were tested, with *P-*values adjusted via Holm–Bonferroni correction. Binary logistic regression served as a sensitivity analysis.

**Results:**

A total of 57.51% of participants were willing to accept OLDU when recommended by a doctor, whereas only 46.44% had understood the definition prior to the study. In the multivariable regression, trust showed the strongest positive association with behavioral intention (B = 0.380, β = 0.377, and *P* < 0.001), followed by facilitating conditions (B = 0.321, β = 0.337, and *P* < 0.001), perceived risk (B = 0.098, β = 0.102, and *P* = 0.002), and social influence (B = 0.091, β = 0.095, and *P* = 0.024). High school/technical secondary education and prior understanding of OLDU were also associated with higher behavioral intention. Subgroup analyses revealed no robust evidence of effect modification after Holm–Bonferroni correction. In the sensitivity analysis, perceived risk remained positively associated with high OLDU acceptance (OR = 1.925 and *P* = 0.027).

**Conclusion:**

The findings highlight the importance of trust and facilitating conditions in OLDU acceptance. The positive association between perceived risk and behavioral intention indicates that recognition of potential risks and willingness to accept treatment are not necessarily mutually exclusive in this clinical decision-making context, although the explanation for this association remains uncertain. This study supports the utility of the extended UTAUT model for understanding high-risk medical decisions and underscores the importance of trust-building and transparent benefit–risk communication in clinical practice.

## 1 Introduction

Off-label drug use (OLDU) involves prescribing medications outside their regulatory-approved indications, a practice justified by evidence-based therapeutic benefits for specific conditions (Zuo et al., 2022). As a necessary and appropriate clinical strategy, OLDU has become an important component of legitimate medical practice worldwide (Ngcobo and Mathibe, 2024). It holds particular significance in obstetrics, a field historically characterized by the exclusion of pregnant women from clinical trials and a consequently limited arsenal of approved medications. Notably, only four drugs targeting pregnancy-related complications, namely, brexanolone, zuranolone, atosiban, and heat-stable carbetocin, have reached the market in the past 3 decades (Makama et al., 2025). This profound drug drought in maternal health has made OLDU not only inevitable but also commonplace in obstetric practice, with applications spanning conditions such as preterm birth, postpartum hemorrhage, pre-eclampsia, and eclampsia (Ammerdorffer et al., 2024).

While OLDU provides a therapeutic alternative when supported by emerging evidence, its risk–benefit profile may not be adequately substantiated by robust clinical-trial data (Chansky et al., 2022). This unapproved drug use not only increases the risk of adverse drug reactions but also raises concerns about medical liability and patient belief (Wittich, Burkle, and Lanier, 2012). The risk–benefit calculus is further complicated in pregnancy, where interventions inherently affect both the mother and the fetus. Consequently, OLDU in this population demands exceptionally high standards of clinical management, requiring decisions grounded in strong scientific evidence and an emphasis on shared decision-making with fully informed patients. However, obtaining adequate informed consent for OLDU remains challenging in obstetric–gynecologic practice, with evidence indicating that over 75% of OLDU prescriptions are issued without consent (Shakeel et al., 2022). This communication gap highlights the critical need to understand pregnant women’s perspectives on OLDU, as their acceptance directly influences treatment adherence and safety. However, research systematically investigating pregnant women’s acceptance of OLDU and its key determinants remains markedly limited.

The complexity of OLDU decision-making in pregnancy necessitates a robust theoretical framework to identify and analyze the multifactorial influences on acceptance. The Unified Theory of Acceptance and Use of Technology (UTAUT), developed by Venkatesh et al. through the integration of eight relevant technology acceptance theories, provides such a framework (Venkatesh et al., 2003). Its core constructs comprise performance expectancy (PE), effort expectancy (EE), social influence (SI), and facilitating conditions (FC) (Ammenwerth, 2019). As the most effective model for investigating technology acceptance, the UTAUT can account for up to 70% of the variance in user intention (Venkatesh et al., 2003). Its applicability has been validated in studies examining the adoption of various healthcare technologies (Wutz et al., 2023; Eiskjær et al., 2023). An extended UTAUT model that includes perceived risk (PR) and trust (TR) is particularly applicable to the context of OLDU. This is because the decision to accept OLDU may be influenced by an evaluation of both its perceived benefits, ease of use, social pressures, and contextual support, along with the individual’s assessment of risks and trust in the healthcare system.

Therefore, in this study, we utilize an extended UTAUT model to understand pregnant women’s perspectives on OLDU and identify the key determinants of their acceptance. The aim is for the findings to inform the development of clinical communication strategies and management guidelines for OLDU during pregnancy.

## 2 Materials and methods

### 2.1 Study design and participants

From March to August of 2025, a cross-sectional survey was conducted among pregnant women attending the outpatient obstetric clinic of the First People’s Hospital of Yulin. The clinic serves patients from multiple regions across Guangxi. Participants were recruited by convenience sampling. The survey was administered by trained obstetricians and clinical pharmacists, who explained the study’s purpose, procedures, and confidentiality measures to potential participants. Before accessing the questionnaire, participants reviewed the study information and provided electronic informed consent by selecting the consent confirmation option on the Wenjuanxing platform (https://www.wjx.cn/). Only participants who confirmed their consent could proceed to and anonymously complete the questionnaire. The questionnaire was designed based on the extended UTAUT model. The study protocol received approval from the Ethics Committee of the First People’s Hospital of Yulin (Approval No: YLSY-IRB-SR-2024064) and adhered to the principles of the Declaration of Helsinki and relevant data-protection regulations.

#### 2.1.1 Inclusion and exclusion criteria

The inclusion criteria were: (1) age ≥18 years, (2) clinically confirmed pregnancy, (3) no severe cognitive impairment or mental illness, (4) ability to complete the questionnaire independently or with guidance, and (5) consent to participate. The exclusion criteria were: (1) severe mental illness or cognitive impairment that prevented completion of the questionnaire, (2) unstable or severe pregnancy-related complications, and (3) refusal to participate.

#### 2.1.2 Sample size estimation

Sample size estimation was based on the criterion of 10 participants per questionnaire item (Liu, 2010). With 21 items used to assess behavioral intention (BI) and six predictor constructs, a minimum of 210 participants was required. Accounting for a 20% non-response rate, we aimed to recruit 263 participants. In total, 512 questionnaires were distributed, yielding 506 valid responses, which was a sufficient number for the planned analyses.

### 2.2 Theoretical framework and variable definitions

The extended UTAUT served as the foundational framework in this study. BI was modeled as being predicted by six constructs: PE, EE, SI, FC, PR, and TR. Definitions of these key variables are provided in Table 1 (Wutz et al., 2023; Eiskjær et al., 2023; Chao, 2019; Wang et al., 2021; Patil and Undale, 2023; Fitrianie et al., 2021; Xu and Xiong, 2026; AlMojaibel et al., 2025; von Winckelmann et al., 2025; Bai and Guo, 2022; Venkatesh, Thong, and Xu, 2016; Tariq et al., 2024), and the conceptual research model is illustrated in Figure 1.

**TABLE 1:** Definitions of key constructs.

Model construct	Definition	References
Performance expectancy	Pregnant women’s expectations regarding the effectiveness and benefits of off-label drug use, including positive outcomes such as health improvement and symptom relief	Eiskjær et al., 2023 Chao, 2019
Effort expectancy	Pregnant women’s perception of the ease or difficulty in obtaining, understanding, and appropriately using information related to off-label drug use	Wang et al., 2021 Patil and Undale, 2023
Social influence	The support or influence from individuals in a pregnant woman’s social environment (e.g., family, physicians, and peers) regarding her medication behavior	Fitrianie et al., 2021 Xu and Xiong, 2026
Facilitating conditions	The accessibility and completeness of medical resources, professional consultation, and relevant information for pregnant women	AlMojaibel et al., 2025 von Winckelmann et al., 2025
Perceived risk	Pregnant women’s concerns about potential adverse consequences of off-label drug use, such as fetal abnormalities or side effects	Wutz et al., 2023 Bai and Guo, 2022
Trust	The degree of confidence pregnant women place in the reliability and safety of healthcare providers, medication-related information, and the clinical environment	Venkatesh et al., 2003 Tariq et al., 2024

**FIGURE 1:**
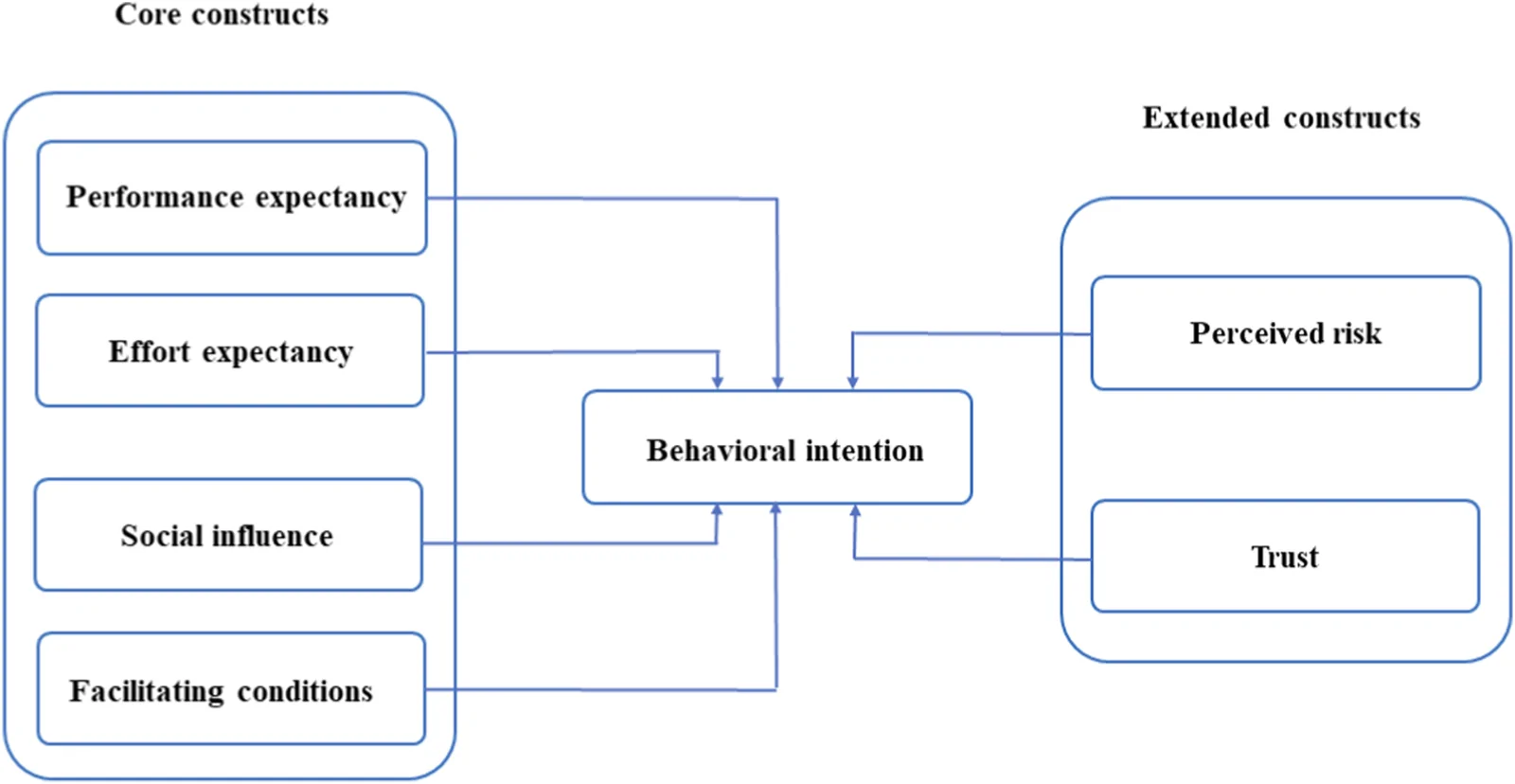
A model for the acceptance of off-label drug use in pregnant women.

### 2.3 Questionnaire design and validation

The initial items were developed based on the seven dimensions: PE, EE, SI, FC, PR, TR, and BI. The draft questionnaire, including its structure and all the items, was reviewed for clinical relevance and appropriateness by two obstetricians and two clinical pharmacists. Since the concept of OLDU might be unfamiliar to some participants, a concise definition was provided preceding the sections on predictor construct measurements and BI. This step ensured a common baseline understanding before respondents proceeded to the main survey modules.

The questionnaire was administered electronically via the Wenjuanxing platform, configured to allow responses only through WeChat accounts. Each WeChat account was restricted to a single submission to prevent duplicate entries. All survey items were mandatory to minimize missing data. To refine the instrument, a pilot survey was conducted with 30 pregnant women. Their feedback on item clarity was used to revise ambiguous wording. Preliminary reliability analysis on the pilot data yielded acceptable Cronbach’s α values for all constructs: PE = 0.740, EE = 0.836, SI = 0.795, FC = 0.928, PR = 0.866, TR = 0.801, and BI = 0.849. The overall scale reliability was excellent (Cronbach’s α = 0.946). Since all values exceeded 0.70 and no items required deletion, the questionnaire was finalized for the main study (Supplementary Table S1).

The final questionnaire consisted of three modules.

1. Basic information (11 items): This section collected demographic and clinical data, including age, gestational trimester, and education level.
2. Measurement of predictor constructs (18 items): The six predictor constructs (PE, EE, SI, FC, PR, and TR) were measured, and each were assessed by three items.
3. BI (three items): This module assessed pregnant women’s acceptance willingness toward OLDU, which was measured as a composite of their intention to accept, commit to adhere, and provide feedback (Table 2).

**TABLE 2:** Measurement items for each construct of off-label drug use in pregnant women.

Construct	Item number	Measurement items
Performance expectancy (PE)	PE1	Off-label drug use can effectively control diseases during pregnancy
	PE2	Off-label drug use can better protect the health of the fetus
	PE3	The potential benefits of off-label drug use for pregnant women and their fetuses outweigh the risks
Effort expectancy (EE)	EE1	I can easily understand the doctor’s explanation about off-label drug use
	EE2	It is easy for me to find authoritative and reliable information about off-label drug use
	EE3	Following the off-label drug use plan as directed by my doctor is easy for me
Social influence (SI)	SI1	The advice of doctors or pharmacists is important in my decision to use off-label drugs
	SI2	Positive public feedback of off-label drug use during pregnancy would increase my willingness to try it
	SI3	Family support would make me more willing to accept off-label drug use
Facilitating conditions (FC)	FC1	Providing detailed educational materials on off-label drug use would help me understand the information easily
	FC2	The medical staff can address my questions about off-label drug use promptly
	FC3	The hospital can coordinate the prenatal examination process to monitor the effectiveness of off-label drug use
Perceived risk (PR)	PR1	I am concerned that off-label drug use may adversely affect fetal health
	PR2	I worry that my rights may not be adequately protected if medical disputes arise from off-label drug use
	PR3	I am concerned that off-label drug use during pregnancy may lead to unexpected side effects
Trust (TR)	TR1	The doctor recommends off-label drug use for the health of both myself and the fetus
	TR2	I have confidence in the hospital’s management of off-label drug use
	TR3	I believe that the doctor will truthfully and accurately inform me of the risks and benefits associated with off-label drug use
Behavioral intention (BI)	BI1	I am willing to accept off-label drug use if recommended by a doctor
	BI2	I will strictly adhere to the dosage and administration requirements for off-label drug use
	BI3	I will proactively report physical reactions to my doctor after using off-label drugs

All items were rated on a five-point Likert scale (1 = strongly disagree; 5 = strongly agree). Higher scores reflected a stronger perception of that construct.

### 2.4 Statistical analysis

Reliability was assessed using Cronbach’s α, with values > 0.70 considered acceptable. Structural validity was evaluated by confirmatory factor analysis using goodness-of-fit index (GFI), adjusted goodness-of-fit index (AGFI), normed fit index (NFI), comparative fit index (CFI), incremental fit index (IFI), and root mean square error of approximation (RMSEA). Convergent validity was supported when composite reliability (CR) ≥ 0.7 and average variance extracted (AVE) ≥ 0.50. The Fornell–Larcker criterion was used to test discriminant validity. It is supported when the square root of each construct’s AVE exceeds its correlations with all other constructs (Alharbi, 2026).

Descriptive statistics were used to summarize demographic information, construct scores, and distributions of attitudes. Categorical variables are presented as frequencies (percentages), while continuous variables are represented by medians (interquartile ranges). Spearman’s rank correlation was used to evaluate the associations between the six UTAUT construct scores and BI. Mann–Whitney U or Kruskal–Wallis tests were used to compare BI based on demographic and clinical factors. Characteristics associated with BI at *P* < 0.05 were considered candidate covariates.

Multiple linear regression was used to examine factors independently associated with BI. All six continuous UTAUT construct scores and characteristics associated with BI in the univariable analyses (*P* < 0.05) were entered simultaneously using the Enter method. Multicollinearity was assessed using variance inflation factors (VIFs), with values < 5 considered acceptable. As a sensitivity analysis, BI was dichotomized at the sample median into high- and low-acceptance groups and analyzed using binary logistic regression. The six UTAUT construct scores were retained as continuous variables, and characteristics associated with the binary outcome in univariable chi-square tests (*P* < 0.05) were selected as covariates. The same Enter method was used.

Exploratory subgroup analyses evaluated effect modification by age, education level, employment status, gestational trimester, drug history during pregnancy, and knowledge of OLDU before the research. Perceived-risk-by-subgroup interactions were evaluated in separate full-sample adjusted linear regression models using joint partial F tests. The interaction *P*-values were adjusted using the Holm–Bonferroni correction. Analyses were performed using IBM SPSS Statistics 23.0, IBM SPSS Amos 23.0, SmartPLS 4, and Python 3.11. All tests were two-sided, with *P* < 0.05 considered statistically significant. Interaction tests were interpreted using Holm-adjusted *P-*values.

## 3 Results

### 3.1 Characteristics of participants​

A total of 506 pregnant women were included in the study. Of them, 45.26% were in the 18–29 years age range. A total of 41.90% of the participants had a bachelor’s degree or higher. The majority of participants (73.12%) reported using drugs during pregnancy; however, less than half (46.44%) of them had understood the definition of OLDU prior to the research. Regarding healthcare utilization, 70.75% primarily sought antenatal care at municipal or provincial general hospitals. Detailed demographic characteristics are presented in Table 3.

**TABLE 3:** Basic characteristics of the 506 pregnant women.

Characteristic	Category	n	%
Age (years)	18–29	229	45.26
	30–39	200	39.53
	≥40	77	15.22
Monthly income (CNY)	<2,000	82	16.21
	2,000–4,000	186	36.76
	4,000–6,000	128	25.30
	>6,000	110	21.74
Residence	Urban	289	57.11
	Rural	217	42.89
Education level	Junior high school or below	135	26.68
	High school/technical secondary school	65	12.85
	Junior college	94	18.58
	Bachelor’s degree or above	212	41.90
Employment status	Healthcare industry	121	23.91
	Non-healthcare industry	249	49.21
	Unemployed	136	26.88
Gestational trimester	<12 weeks	144	28.46
	12–28 weeks	155	30.63
	>28 weeks	207	40.91
Parity	0 (primipara)	188	37.15
	1	203	40.12
	2	67	13.24
	≥3	48	9.49
History of assisted reproduction	Yes	43	8.50
	No	463	91.50
Hospital level for main antenatal care	Municipal or provincial general hospitals	358	70.75
	District/county-level hospital	51	10.08
	Community/township health center	23	4.55
	Maternal and child health hospital	57	11.26
	Other	17	3.36
Drug history during pregnancy	Yes	370	73.12
	No	136	26.88
Knowledge of off-label drug use before the research	Completely unaware	119	23.52
	Heard of but unclear about the concept	152	30.04
	Understood the definition	235	46.44

Abbreviations: n, number.

### 3.2 Reliability and validity test results of the questionnaire

The overall scale demonstrated excellent reliability with a Cronbach’s α of 0.958. Across the seven constructs, Cronbach’s α coefficients ranged from 0.757 to 0.866, all exceeding the threshold of 0.70. Confirmatory factor analysis yielded acceptable model-fit indices: GFI = 0.875, AGFI = 0.828, NFI = 0.903, CFI = 0.922, IFI = 0.923, and RMSEA = 0.084. CR ranged from 0.860 to 0.918, and AVE ranged from 0.672 to 0.789, supporting satisfactory convergent validity (Supplementary Table S2). The square root of each construct’s AVE (0.820–0.888) exceeded its correlations with all other constructs, confirming adequate discriminant validity (Supplementary Table S3).

### 3.3 Descriptive statistics of the predictor constructs and behavioral intention

As shown in Table 4, the median scores for SI, FC, TR, and BI were all 4.0, higher than those for PE (3.7), PR (3.7), and EE (3.3). All constructs exhibited concentrated distributions, with an interquartile range of 3.0–4.0.

**TABLE 4:** Scores of the predictor constructs and behavioral intention.

Construct	Median score	IQR, first quarter–third quarter
Performance expectancy	3.7	3.0–4.0
Effort expectancy	3.3	3.0–4.0
Social influence	4.0	3.0–4.0
Facilitating conditions	4.0	3.0–4.0
Perceived risk	3.7	3.0–4.0
Trust	4.0	3.0–4.0
Behavioral intention	4.0	3.0–4.0

Abbreviation: IQR, interquartile range.

The detailed response distribution for each item is provided in Supplementary Table S4. Figure 2 shows the proportion of responses indicating agreement (combining “agree” and “strongly agree”) for each item. Within facilitating conditions, 64.03% agreed that medical staff could promptly address questions (FC2). TR received the highest endorsement (67.59%, TR3), while EE received the lowest (48.02%, EE2). For PR, over half of the respondents expressed concerns about medical disputes (56.52%, PR2), fetal health (55.53%, PR1), and unexpected side effects (52.57%, PR3). Regarding BI, 57.51% of pregnant women expressed willingness to accept OLDU if recommended by a doctor (BI1).

**FIGURE 2:**
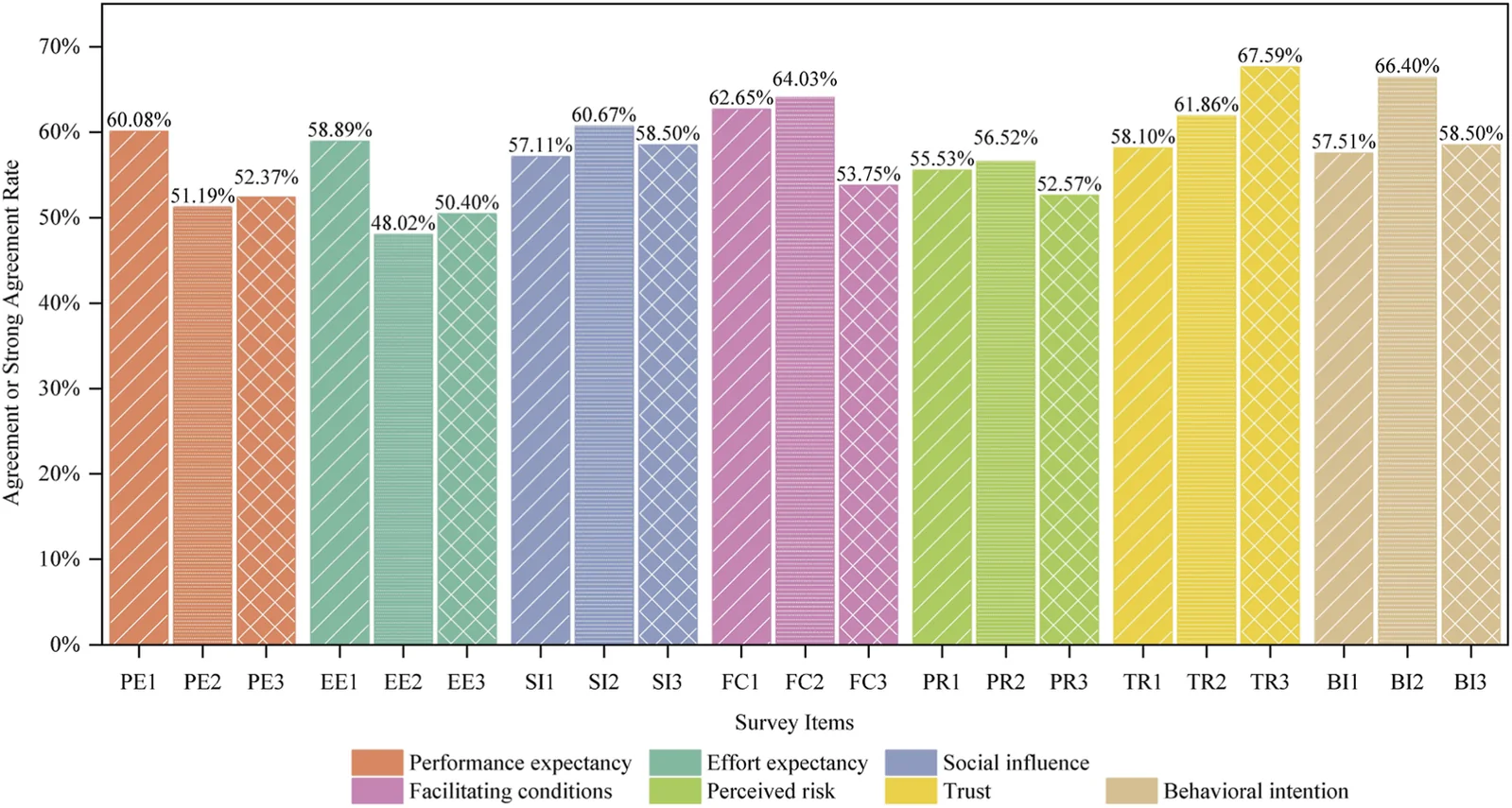
Proportion of responses indicating agreement or strong agreement for each item. PE: performance expectancy; EE: effort expectancy; SI: social influence; FC: facilitating conditions; PR: perceived risk; TR: trust; BI: behavioral intention.

### 3.4 Association analysis results

#### 3.4.1 Associations between predictor constructs and behavioral intention

Table 5 presents the correlations between the factor scores and the BI. All pairwise correlations were positive and statistically significant (*P* < 0.001). Spearman’s correlation coefficients among the predictor constructs ranged from 0.507 to 0.813, with the strongest correlation observed between EE and FC (Spearman’s ρ = 0.813). BI was positively correlated with all constructs (*P* < 0.001). The strongest correlation was observed with FC (ρ = 0.815), followed by TR (ρ = 0.793) and EE (ρ = 0.731).

**TABLE 5:** Correlation between predictor constructs and behavioral intention.

Construct	Performance expectancy	Effort expectancy	Social influence	Facilitating conditions	Perceived risk	Trust	Behavioral intention
Performance expectancy	1.000	​	​	​	​	​	​
Effort expectancy	0.684 *P* < 0.001	1.000	​	​	​	​	​
Social influence	0.710 *P* < 0.001	0.732 *P* < 0.001	1.000	​	​	​	​
Facilitating conditions	0.656 *P* < 0.001	0.813 *P* < 0.001	0.754 *P* < 0.001	1.000	​	​	​
Perceived risk	0.507 *P* < 0.001	0.638 *P* < 0.001	0.592 *P* < 0.001	0.682 *P* < 0.001	1.000	​	​
Trust	0.575 *P* < 0.001	0.678 *P* < 0.001	0.674 *P* < 0.001	0.735 *P* < 0.001	0.652 *P* < 0.001	1.000	​
Behavioral intention	0.581 *P* < 0.001	0.731 *P* < 0.001	0.702 *P* < 0.001	0.815 *P* < 0.001	0.674 *P* < 0.001	0.793 *P* < 0.001	1.000

Values are presented as Spearman’s rank correlation coefficients (ρ) and *P-*values.

#### 3.4.2 Associations between participant characteristics and behavioral intention​

Significant associations with BI were found for age, education level, employment status, gestational trimester, hospital level for main antenatal care, drug history during pregnancy, and prior knowledge of OLDU (*P* < 0.05). No significant associations (*P* > 0.05) were observed with monthly income, residence, parity, and history of assisted reproduction (Table 6).

**TABLE 6:** Univariable comparisons of behavioral intention across demographic and clinical characteristics (n = 506).

Characteristic	Category	n	BI, median (IQR)	U/H	*P-*value
Age (years)	18–29	229	4.00 (3.00–4.00)	37.644	**<0.001**
	30–39	200	4.00 (3.25–4.00)	​	​
	≥40	77	3.00 (2.67–3.67)	​	​
Monthly income (CNY)	<2,000	82	3.67 (3.00–4.00)	2.828	0.419
	2,000–4,000	186	4.00 (3.00–4.00)	​	​
	4,000–6,000	128	4.00 (3.00–4.00)	​	​
	>6,000	110	3.67 (3.00–4.00)	​	​
Residence	Urban	289	4.00 (3.00–4.00)	29,780	0.319
	Rural	217	4.00 (3.00–4.00)	​	​
Education level	Junior high school or below	135	3.33 (3.00–4.00)	23.740	**<0.001**
	High school/technical secondary school	65	4.00 (3.00–4.00)	​	​
	Junior college	94	4.00 (3.00–4.00)	​	​
	Bachelor’s degree or above	212	4.00 (3.00–4.00)	​	​
Employment status	Healthcare industry	121	4.00 (3.00–4.33)	7.173	**0.028**
	Non-healthcare industry	249	3.67 (3.00–4.00)	​	​
	Unemployed	136	4.00 (3.00–4.00)	​	​
Gestational trimester	<12 weeks	144	4.00 (3.00–4.00)	13.679	**0.001**
	12–28 weeks	155	4.00 (3.00–4.17)	​	​
	>28 weeks	207	3.67 (3.00–4.00)	​	​
Parity	0	188	4.00 (3.00–4.00)	2.174	0.537
	1	203	4.00 (3.00–4.00)	​	​
	2	67	4.00 (3.00–4.00)	​	​
	≥3	48	3.50 (3.00–4.00)	​	​
History of assisted reproduction	Yes	43	4.00 (3.00–4.50)	8,344	0.071
	No	463	4.00 (3.00–4.00)	​	​
Hospital level for main antenatal care	Municipal/provincial general hospital	358	4.00 (3.00–4.00)	64.086	**<0.001**
	District/county-level hospital	51	3.00 (3.00–3.67)	​	​
	Community/township health center	23	3.00 (2.83–3.33)	​	​
	Maternal and child health hospital	57	3.00 (2.67–3.67)	​	​
	Other	17	4.00 (3.00–5.00)	​	​
Drug history during pregnancy	Yes	370	4.00 (3.00–4.00)	22,284	**0.042**
	No	136	3.67 (3.00–4.00)	​	​
Knowledge of off-label drug use before the research	Completely unaware	119	3.67 (3.00–4.00)	9.485	**0.009**
	Heard of but unclear about the concept	152	4.00 (3.00–4.00)	​	​
	Understood the definition	235	4.00 (3.00–4.33)	​	​

Abbreviation: BI, behavioral intention; IQR, interquartile range; U, Mann–Whitney U statistic; H, Kruskal–Wallis H statistic. Mann–Whitney U tests were used for two-group comparisons, and Kruskal–Wallis tests for comparisons of three or more groups. Significant associations are shown in bold.

### 3.5 Factors associated with behavioral intention

Table 7 presents the results of the multiple linear regression analysis. The overall model was statistically significant and explained 77.0% of the variance in BI (adjusted *R* ^*2*^ = 0.770). There was no problematic multicollinearity, as all VIFs were less than 5.

**TABLE 7:** Multiple linear regression analysis of factors associated with behavioral intention toward off-label drug use (n = 506).

Variable	B	SE	β	t	*P*-value	95% CI	VIF
UTAUT constructs (continuous)							
Performance expectancy	−0.035	0.032	−0.037	−1.080	0.280	−0.098 to 0.028	2.578
Effort expectancy	0.052	0.037	0.058	1.381	0.168	−0.022 to 0.125	3.837
Social influence	0.091	0.040	0.095	2.264	**0.024**	0.012 to 0.169	3.849
Facilitating conditions	0.321	0.043	0.337	7.435	**<0.001**	0.236 to 0.406	4.518
Perceived risk	0.098	0.031	0.102	3.135	**0.002**	0.036 to 0.159	2.329
Trust	0.380	0.036	0.377	10.515	**<0.001**	0.309 to 0.451	2.827
Age (ref: 18–29 years)							
30–39 years	0.064	0.042	0.036	1.545	0.123	−0.017 to 0.146	1.222
≥40 years	−0.099	0.063	−0.041	−1.572	0.117	−0.223 to 0.025	1.519
Education level (ref: Junior high school or below)							
High school/technical secondary school	0.139	0.067	0.054	2.090	**0.037**	0.008 to 0.270	1.469
Junior college	0.059	0.061	0.027	0.961	0.337	−0.061 to 0.179	1.672
Bachelor’s degree or above	0.107	0.057	0.061	1.867	0.062	−0.006 to 0.219	2.356
Employment status (ref: Healthcare industry)							
Non-healthcare industry	−0.005	0.047	−0.003	−0.098	0.922	−0.097 to 0.088	1.645
Unemployed	−0.001	0.058	−0.001	−0.021	0.983	−0.116 to 0.114	1.984
Gestational trimester (ref: <12 weeks)							
12–28 weeks	−0.033	0.051	−0.018	−0.642	0.521	−0.134 to 0.068	1.651
>28 weeks	−0.081	0.047	−0.046	−1.732	0.084	−0.173 to 0.011	1.560
Hospital level for main antenatal care (ref: Municipal/provincial general hospital)							
District/county-level hospital	−0.081	0.065	−0.028	−1.239	0.216	−0.209 to 0.047	1.144
Community/township health center	0.057	0.095	0.014	0.605	0.545	−0.129 to 0.244	1.153
Maternal and child health hospital	−0.023	0.068	−0.009	−0.341	0.733	−0.158 to 0.111	1.377
Other	0.163	0.107	0.034	1.514	0.131	−0.048 to 0.374	1.105
Medication history during pregnancy (ref: Yes)							
No	0.012	0.044	0.006	0.281	0.779	−0.074 to 0.098	1.107
Knowledge of off-label drug use before the research (ref: Completely unaware)							
Heard of but unclear about the concept	0.019	0.053	0.010	0.358	0.721	−0.084 to 0.122	1.713
Understood the definition	0.115	0.049	0.066	2.343	0.020	0.019 to 0.211	1.757
Adjusted *R* ^*2*^	0.770						
*F*	*F* (22, 483) = 77.94, *P* < 0.001						

Abbreviation: B, unstandardized regression coefficient; β, standardized regression coefficient; CI, confidence interval; SE, standard error; VIF, variance inflation factors. Significant associations are shown in bold.

Higher behavioral intention was independently associated with higher scores for TR (B = 0.380, 95% CI 0.309 to 0.451, β = 0.377, and *P* < 0.001), FC (B = 0.321, 95% CI 0.236 to 0.406, β = 0.337, and *P* < 0.001), PR (B = 0.098, 95% CI 0.036 to 0.159, β = 0.102, and *P* = 0.002), and SI (B = 0.091, 95% CI 0.012 to 0.169, β = 0.095, and *P* = 0.024). PE and EE were not independently associated with BI (*P* > 0.05). Compared with participants with a junior high school education or below, those with a high school/technical secondary education had higher BI (B = 0.139, 95% CI 0.008 to 0.270, and *P* = 0.037). Participants who had understood the definition of OLDU before the survey also had higher BI than those who were completely unaware (B = 0.115, 95% CI 0.019 to 0.211, and *P* = 0.020).

### 3.6 Subgroup and interaction analyses

Figure 3 presents the subgroup-specific associations between PR and BI. A nominal interaction was observed for employment status before multiplicity adjustment (*P* for interaction = 0.026), but it did not remain statistically significant after Holm–Bonferroni correction (Holm-adjusted *P* = 0.154). None of the other interaction tests were significant after correction (Holm-adjusted *P* > 0.05), providing no statistically robust evidence of effect modification.

**FIGURE 3:**
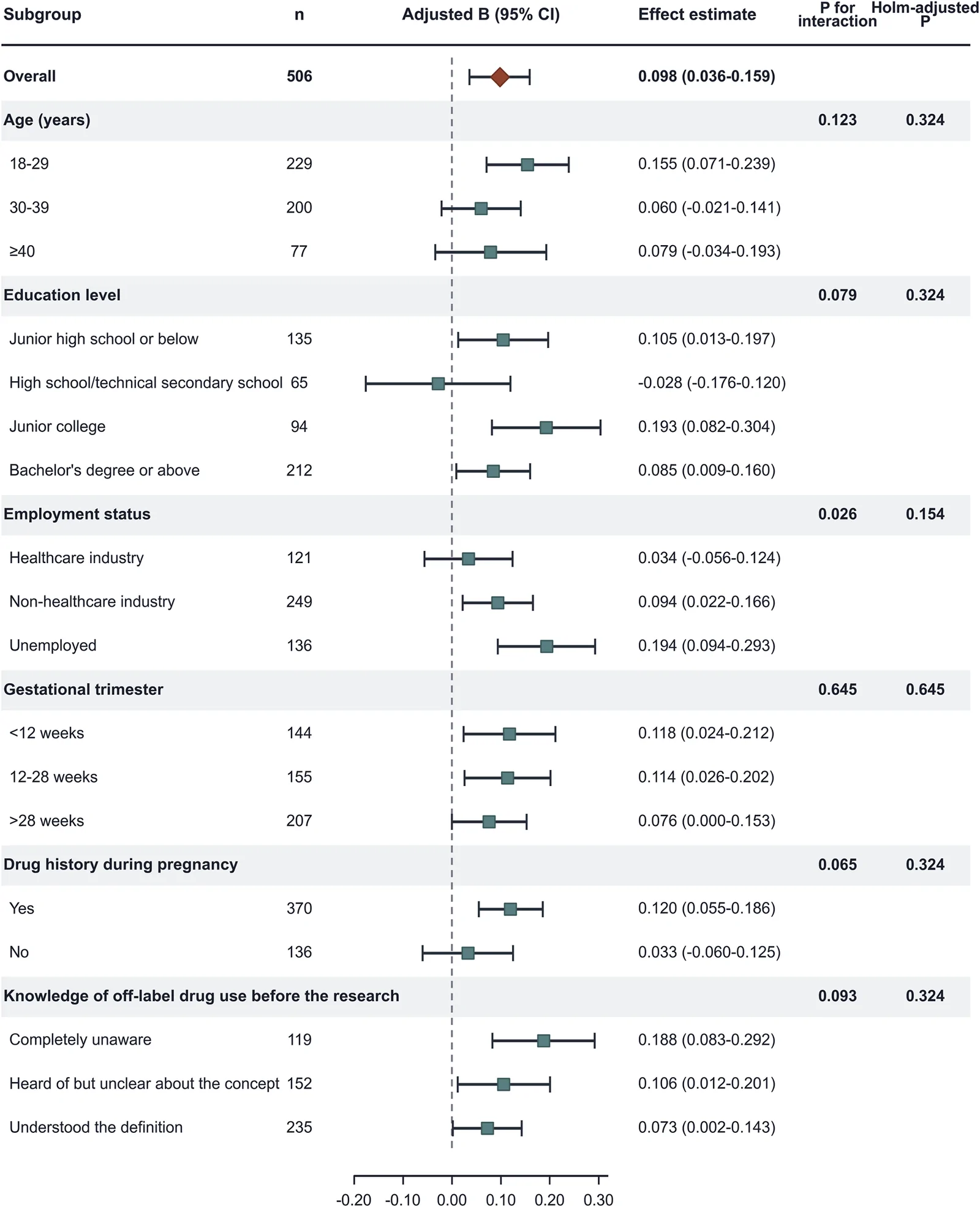
Subgroup analysis of the association between perceived risk and behavioral intention. Squares represent subgroup-specific adjusted unstandardized regression coefficients (B) with 95% CIs, and the diamond represents the overall estimate. Models were adjusted for the other five UTAUT constructs, age, education level, employment status, gestational trimester, hospital level for antenatal care, drug history during pregnancy, and knowledge of off-label drug use before the research. Interaction *P-*values were adjusted using the Holm–Bonferroni procedure. CI, confidence interval.

### 3.7 Sensitivity analysis

In the sensitivity analysis with BI dichotomized at the sample median, univariable chi-square analyses identified age, education level, gestational trimester, hospital level for antenatal care, and drug history during pregnancy as candidate covariates (*P* < 0.05) (Supplementary Table S5). In the binary logistic regression model, PR remained positively associated with high OLDU acceptance (OR = 1.925, 95% CI 1.079 to 3.435, *P* = 0.027), consistent with the positive association observed in the multiple linear regression analysis (Supplementary Table S6).

## 4 Discussion

This study recruited 506 pregnant women from a tertiary hospital in southern China, marking it the first application of the extended UTAUT model to assess willingness to accept OLDU during pregnancy. By incorporating demographic characteristics, we investigated attitudes toward OLDU and identified the key influencing factors.

### 4.1 Attitudes and cognitive characteristics of pregnant women toward OLDU

This study revealed that pregnant women generally held a cautious attitude toward OLDU. The agreement rates for accepting treatment, following medical advice, and providing feedback after administration were 57.51%, 66.40%, and 58.50%, respectively. This finding is consistent with a previous study reporting that only 51% of parents of healthy children accepted OLDU in the absence of alternative treatments (Lenk et al., 2009). These results indicate that vulnerable populations generally exhibit a conservative decision-making tendency regarding OLDU. This further underscores that downplaying or inadequately informing pregnant women about OLDU in clinical practice lacks ethical justification.

The cautious attitude among pregnant women is closely associated with a limited understanding of OLDU, an issue that warrants high clinical priority. In this study, we found that only 46.44% of pregnant women had understood the definition of OLDU, with nearly 60% of respondents exhibiting cognitive gaps or a vague understanding. Given that 60% of WHO essential medicines have at least one documented off-label use (Kateel, Hegde, and Holla, 2025), physicians may neglect to fully inform pregnant women due to long-standing prescribing habits, thereby exacerbating cognitive biases. Furthermore, only 48.02% of pregnant women could conveniently access reliable information on OLDU, and the score of EE was the lowest among all constructs, indicating that pregnant women face substantial difficulties in obtaining OLDU-related information. Inadequate knowledge of OLDU may impair pregnant women’s ability to make balanced risk–benefit judgments, making it a critical factor influencing medication adherence.

### 4.2 Key factors associated with behavioral intention

This study identified the key factors of intention to accept OLDU among pregnant women. TR was the strongest predictor, with higher trust levels significantly and positively associated with greater acceptance. This result supports the notion that individual trust determines behaviors and acceptance (Siau and Wang, 2018) and aligns with recent UTAUT-based studies on healthcare technology adoption. Studies on the intention to use medical artificial intelligence (AI) among healthcare practitioners have also identified trust as the strongest predictor of acceptance (Dingel et al., 2024; Dai et al., 2025). This signifies that trust is a key psychological boundary condition for technology adoption in high-uncertainty medical decision-making contexts (Ju et al., 2026). Trust is essential to healthcare delivery, as evidenced by its positive impact on treatment compliance (Gille, Smith, and Mays, 2015). For pregnant women, trust in healthcare providers may help mitigate concerns about the uncertainty of OLDU, thereby improving adherence to such treatment regimens.

PR is determined by both the unpredictability of outcomes and perceived intensity (Baker, 2001). In this study, PR was defined as pregnant women’s expectations of adverse outcomes from OLDU. We found that PR was positively associated with BI, a finding that contradicts the fundamental assumption of traditional technology acceptance models that risk serves as a behavioral barrier. It also differs from studies in which PR reduced or did not affect BI (Dai et al., 2025; Yao et al., 2023; Zhao et al., 2025; Ye et al., 2025).

Unlike voluntary technology adoption, OLDU during pregnancy is a clinician-mediated medical decision that involves the simultaneous consideration of maternal–fetal benefits and harms. Previous research has shown that pregnant women weigh both medication-related risks and the potential consequences of untreated disease, calling for adequate information or counseling on medication safety to guide their decisions (Lynch et al., 2018; Mulder et al., 2018). Therefore, awareness of potential risks does not necessarily preclude willingness to consider clinically indicated treatment, particularly when professional guidance and support are available. This interpretation is consistent with the DASH-TOP study, in which pregnant women acknowledged the burden and uncertainty associated with prophylactic low-molecular-weight heparin but rated daily LMWH injections as the best state closest to a healthy pregnancy (León-García et al., 2024). This finding indicates that recognition of potential risks and willingness to accept treatment are not necessarily mutually exclusive in the clinical decision-making context. However, because benefit–risk deliberation and temporal ordering were not directly assessed, the mechanism underlying the observed positive association remains uncertain. This highlights the need for clear and balanced benefit–risk communication in clinical practice and for longitudinal studies to investigate the association further. Exploratory subgroup analyses found that the positive association between PR and BI was not driven by any single demographic subgroup. The exploratory subgroup analyses provided no statistically robust evidence that the PR–BI association differed across the characteristics examined. Thus, the available data did not support clear subgroup-specific differences in this association.

Facilitating conditions also showed a positive association with intention to accept OLDU, consistent with previous studies demonstrating that easy access to information and sufficient professional support significantly improve acceptance (Breitwieser et al., 2025; Fan et al., 2025). Therefore, optimizing professional consultation, reliable information, and medication monitoring may reduce the informational and practical burdens involved in OLDU decisions. Subjective norms shaped by the influence of others have been shown to affect health behavior in pregnancy-specific contexts (Kim, Kang, and Kim, 2025; Singh et al., 2024), and similar social dynamics may also operate in OLDU decision-making. In the present study, SI was independently associated with BI. Although the association was relatively modest, it nonetheless indicates that the opinions and recommendations of clinicians, family members, or other individuals may shape treatment decisions. PE is one of the original UTAUT constructs (Venkatesh et al., 2003). However, in the context of OLDU during pregnancy, expected treatment benefits may be weighed alongside potential maternal–fetal risks, trust in healthcare professionals, and available clinical support rather than considered in isolation. Thus, the absence of an independent association between PE and BI does not imply that expected treatment benefits are unimportant. Instead, these benefits may be considered within a broader benefit–risk evaluation.

Compared with participants with a junior high school education or below, higher BI was observed only among those with a high school/technical secondary education, whereas no statistically significant differences were found for junior college, bachelor’s degree, or above. Education may facilitate access to and evaluation of medication-related information, as educational attainment has been associated with medication information literacy among pregnant women (Zhang et al., 2021). However, higher educational attainment does not necessarily translate into greater willingness to use medication during pregnancy. Previous studies have found that less educated pregnant women tend to believe that medications in general are harmful, while highly educated women may be more reluctant to use medicines or have a higher threshold for medication use (Nordeng, Koren, and Einarson, 2010; Ceulemans et al., 2019). Thus, the relationship between education and OLDU acceptance may be complex and context-dependent rather than graded.

Prior understanding of the definition of OLDU was associated with higher BI. Awareness of this concept may reduce unfamiliarity and uncertainty surrounding OLDU and help pregnant women participate more effectively in discussions about the treatment rationale. Previous research has shown that pregnant women commonly have misconceptions and unmet information needs regarding medication use (Kirubarajan et al., 2021), whereas pregnancy-specific medication education can improve medication information literacy and decision self-efficacy (Li et al., 2025), providing indirect support for this interpretation. Although self-reported understanding of the definition does not necessarily indicate comprehensive knowledge, this finding highlights the importance of clearly explaining the concept of OLDU and the associated benefit–risk information to support informed treatment decisions.

### 4.3 Implications of the study

This study provides insights for both clinical practice and theoretical advancement. From a clinical perspective, medical institutions should establish standardized and individualized procedures for communicating about OLDU. To promote trust between physicians and patients, professional and transparent communication needs to be strengthened. In addition, diverse channels should be developed to provide pregnant women with access to reliable information, enabling them to make autonomous decisions. In theory, this study is the first to use the extended UTAUT model to analyze medication acceptance intentions among pregnant women, demonstrating the model’s usefulness in high-risk medical decisions. We confirmed the central role of TR and observed a positive association between PR and acceptance intention, providing a valuable supplement to the traditional technology acceptance model.

### 4.4 Limitations and future research directions

We acknowledge some limitations of the present study. First, the study participants were recruited exclusively from a single tertiary hospital in China. The single-center design may introduce biases related to geography, hospital level, and population demographics, hence limiting the applicability of the findings to wider groups. Second, this study primarily relied on self-reported data from pregnant women, which could be skewed by social desirability and recollection bias. We were unable to fully exclude these biases even though we informed participants that their answers would be kept fully private and would not have an impact on their clinical care. Third, several potential influencing factors on OLDU willingness, such as pregnancy caregiver involvement, disease diagnoses, and class polypharmacy, were not analyzed and should be addressed in future research.

Future multi-center and large-sample studies may be conducted to improve the representativeness of the results. Meanwhile, interventional studies could explore the effects of intervention strategies based on risk communication, trust-building, and stratified management on pregnant women’s acceptance of OLDU. This would provide higher-level evidence for clinical practice.

## 5 Conclusion

The findings highlight the importance of TR and facilitating conditions in OLDU acceptance. The positive association between PR and BI indicates that recognition of potential risks and willingness to accept treatment are not necessarily mutually exclusive in this clinical decision-making context, although the explanation for this association remains uncertain. This study supports the utility of the extended UTAUT model for understanding high-risk medical decisions and underscores the importance of trust-building and transparent benefit–risk communication in clinical practice.

## Data Availability

The original contributions presented in the study are included in the article/Supplementary Material further inquiries can be directed to the corresponding authors.
