# Supplementary material for: Determinants of off-label drug acceptance in pregnancy: insights from an extended UTAUT model
Source: Front Pharmacol. 2026 Sep 21;17:1850058. doi: 10.3389/fphar.2026.1850058 (PMC13635127; doi:10.3389/fphar.2026.1850058)
Supplement: Supplementary file 1 [file Table1.docx]

|  | **Supplementary Table S1.** Survey questionnaire on pregnant women’s attitudes and acceptance of off-label drug use. | | |
| --- | --- | --- | --- |
|  | Dear Madam,  We are currently conducting a survey to assess pregnant women's attitudes and behavioral intentions towards off-label drug use. The aim is to explore the key factors influencing pregnant women's acceptance of off-label drug use and to provide a scientific basis for optimizing medication guidance during pregnancy.  1. Study content  This study employs an anonymous questionnaire survey. The content covers: your basic demographic information (e.g., age and education level) and your attitudes and behavioral intentions regarding off-label drug use during pregnancy.  2. Voluntary participation  Your participation is entirely voluntary. You have the right to withdraw from the survey at any time, and this will not affect your medical care or treatment.  3. Privacy protection  The questionnaire is completed anonymously. All data will be used solely for statistical analysis, and your personal information will not be disclosed. The research findings will be presented in aggregated form, and no individual responses will be reported.  4. Contact information  If you have any questions about the study, please feel free to contact us.  Principal investigator: Liang Chunhong  Institution: The First People's Hospital of Yulin  Telephone: 0775-2683190  Email: lchhon@163.com  □I have been informed and agree that the collected data will be used for scientific research. | | |
| **Part 1 Basic information** | | |  |
| **1. Your age (years)：** | | a. 18-29  b. 30-39  c. ≥40 |  |
| 2. **Monthly income (CNY):** | | a. <2000  b. 2000-4000  c. 4000-6000  d. >6000 |  |
| 3. **place of residence：** | | a. Urban  b. Rural |  |
| 4. **Education level：** | | a. Junior high school or below b. High school/technical secondary school c. Junior college d. Bachelor's degree or above |  |
| **5. Employment status：** | | a. Healthcare industry  b. Non-healthcare industry  c. Unemployed |  |
| **6.** **Gestational trimester:** | | a. <12 weeks  b. 12-28 weeks  c. >28 weeks |  |
| **7.** **Parity：** | | a. 0 (primipara)  b. 1  c. 2  d. ≥3 |  |
| **8.** **History of assisted reproduction:** | | a.Yes  b. No |  |
| **9.** **Hospital level for main antenatal care：** | | a. Municipal or provincial general hospitals  b. District/county-level hospital  c. Community/township health center  d. Maternal and child health hospital  e. Other |  |
| **10.** **Drug history during pregnancy:** | | a. Yes  b. No |  |
| **11.** **Knowledge of off-label drug use before the research:** | | a. Completely unaware  b. Heard of but unclear about the concept  c. Understood the definition |  |

| **Part 2 Attitudes and behavioral intention toward off-label drug use during pregnancy**  ^*^Before answering the following questions, please read the following definition and legal basis for off-label drug use:   1. "Off-label drug use" refers to the use of a medication for indications, dosages, or patient populations that are not included in the officially approved drug information sheet.   2. According to the "Physician Law of the People's Republic of China": Under special circumstances where there is no effective or better treatment available, and after obtaining the patient's explicit informed consent, physicians may use a drug in a manner not specified in the drug information sheet, provided that there is evidence-based medical support for such use. | | | | | |
| --- | --- | --- | --- | --- | --- |
| 1. **Off-label drug use can effectively control diseases during pregnancy.** | a. strongly disagree | b. disagree | c. neutral | d. agree | e. strongly agree |
| 2. **Off-label drug use can better protect the health of fetus.** | a. strongly disagree | b. disagree | c. neutral | d. agree | e. strongly agree |
| 3. **The potential benefits of off-label drug use for pregnant women and their fetuses outweigh the risks.** | a. strongly disagree | b. disagree | c. neutral | d. agree | e. strongly agree |
| 4. **I can easily understand the doctor's explanation about off-label drug use.** | a. strongly disagree | b. disagree | c. neutral | d. agree | e. strongly agree |
| 5. **It is easy for me to find authoritative and reliable information about off-label drug use.** | a. strongly disagree | b. disagree | c. neutral | d. agree | e. strongly agree |
| 6. **Following the off-label drug use plan as directed by my doctor is easy for me.** | a. strongly disagree | b. disagree | c. neutral | d. agree | e. strongly agree |
| 7. **The advice of doctors or pharmacists is important in my decision to use off-label drugs.** | a. strongly disagree | b. disagree | c. neutral | d. agree | e. strongly agree |
| 8. **Positive public feedback of off-label drug use during pregnancy would increase my willingness to try it.** | a. strongly disagree | b. disagree | c. neutral | d. agree | e. strongly agree |
| 9. **Family support would make me more willing to accept off-label drug use.** | a. strongly disagree | b. disagree | c. neutral | d. agree | e. strongly agree |
| 10. **Providing detailed educational materials on off-label drug use would help me understand the information easily.** | a. strongly disagree | b. disagree | c. neutral | d. agree | e. strongly agree |
| 11. **The medical staff can address my questions about off-label drug use promptly.** | a. strongly disagree | b. disagree | c. neutral | d. agree | e. strongly agree |
| 12. **The hospital can coordinate the prenatal examination process to monitor the effectiveness of off-label drug use.** | a. strongly disagree | b. disagree | c. neutral | d. agree | e. strongly agree |
| 13. **I am concerned that off-label drug use may adversely affect fetal health.** | a. strongly disagree | b. disagree | c. neutral | d. agree | e. strongly agree |
| 14. **I worry that my rights may not be adequately protected if medical disputes arise from off-label drug use.** | a. strongly disagree | b. disagree | c. neutral | d. agree | e. strongly agree |
| 15. **I am concerned that off-label drug use during pregnancy may lead to unexpected side effects.** | a. strongly disagree | b. disagree | c. neutral | d. agree | e. strongly agree |
| 16. **The doctor recommends off-label drug use for the health of both myself and the fetus.** | a. strongly disagree | b. disagree | c. neutral | d. agree | e. strongly agree |
| 17. **I have confidence in the hospital's management of off-label drug use.** | a. strongly disagree | b. disagree | c. neutral | d. agree | e. strongly agree |
| 18. **I believe that the doctor will truthfully and accurately inform me of the risks and benefits associated with off-label drug use.** | a. strongly disagree | b. disagree | c. neutral | d. agree | e. strongly agree |
| 19. **I am willing to accept off-label drug use if recommended by a doctor.** | a. strongly disagree | b. disagree | c. neutral | d. agree | e. strongly agree |
| 20. **I will strictly adhere to the dosage and administration requirements for off-label drug use.** | a. strongly disagree | b. disagree | c. neutral | d. agree | e. strongly agree |
| 21. **I will proactively report physical reactions to my doctor after using off-label drugs.** | a. strongly disagree | b. disagree | c. neutral | d. agree | e. strongly agree |
