# Supplementary material for: Determinants of off-label drug acceptance in pregnancy: insights from an extended UTAUT model
Source: Front Pharmacol. 2026 Sep 21;17:1850058. doi: 10.3389/fphar.2026.1850058 (PMC13635127; doi:10.3389/fphar.2026.1850058)
Supplement: Supplementary file 2 [file Table2.docx]

**Supplementary Table S2.** Reliability and convergent validity of the measurement model.

| **Construct** | **Item** | **Standardized outer loading** | **Cronbach's α** | **CR** | **AVE** |
| --- | --- | --- | --- | --- | --- |
| Performance expectancy | Q1 | 0.813 | 0.809 | 0.886 | 0.721 |
|  | Q2 | 0.876 |  |  |  |
|  | Q3 | 0.858 |  |  |  |
| Effort expectancy | Q4 | 0.823 | 0.841 | 0.905 | 0.760 |
|  | Q5 | 0.892 |  |  |  |
|  | Q6 | 0.897 |  |  |  |
| Social influence | Q7 | 0.852 | 0.770 | 0.866 | 0.683 |
|  | Q8 | 0.799 |  |  |  |
|  | Q9 | 0.828 |  |  |  |
| Facilitating conditions | Q10 | 0.875 | 0.866 | 0.918 | 0.789 |
|  | Q11 | 0.875 |  |  |  |
|  | Q12 | 0.914 |  |  |  |
| Perceived risk | Q13 | 0.864 | 0.802 | 0.883 | 0.717 |
|  | Q14 | 0.811 |  |  |  |
|  | Q15 | 0.863 |  |  |  |
| Trust | Q16 | 0.836 | 0.757 | 0.860 | 0.672 |
|  | Q17 | 0.767 |  |  |  |
|  | Q18 | 0.854 |  |  |  |
| Behavioral intention | Q19 | 0.854 | 0.809 | 0.887 | 0.724 |
|  | Q20 | 0.834 |  |  |  |
|  | Q21 | 0.864 |  |  |  |

Abbreviations: CR, composite reliability; AVE, average variance extracted.

**Supplementary Table S3.** Discriminant validity assessed using the Fornell-Larcker criterion.

| Construct | Behavioral intention | Effort expectancy | Facilitating conditions | Performance expectancy | Perceived risk | Social influence | Trust |
| --- | --- | --- | --- | --- | --- | --- | --- |
| Behavioral intention | **0.851** |  |  |  |  |  |  |
| Effort expectancy | 0.720 | **0.872** |  |  |  |  |  |
| Facilitating conditions | 0.808 | 0.808 | **0.888** |  |  |  |  |
| Performance expectancy | 0.593 | 0.690 | 0.666 | **0.849** |  |  |  |
| Perceived risk | 0.680 | 0.642 | 0.690 | 0.526 | **0.846** |  |  |
| Social influence | 0.723 | 0.763 | 0.793 | 0.732 | 0.619 | **0.827** |  |
| Trust | 0.797 | 0.671 | 0.738 | 0.588 | 0.681 | 0.712 | **0.820** |

Diagonal values in bold represent the square roots of AVE; off-diagonal values are latent-variable correlations estimated using SmartPLS.

**Supplementary Table S4.** Response distribution for the predictor constructs and behavioral intention.

| **Construct** | **Item**  **number** | **Strongly disagree n(%)** | **Disagree n(%)** | **Neutral n(%)** | **Agree n(%)** | **Strongly agree n(%)** | **Agree/Strongly agree**  **n(%)** |
| --- | --- | --- | --- | --- | --- | --- | --- |
| Performance expectancy | PE1 | 29(5.73) | 48(9.49) | 125(24.70) | 201(39.72) | 103(20.36) | 304(60.08) |
|  | PE2 | 51(10.08) | 27(5.34) | 169(33.40) | 183(36.17) | 76(15.02) | 259(51.19) |
|  | PE3 | 33(6.52) | 33(6.52) | 175(34.58) | 189(37.35) | 76(15.02) | 265(52.37) |
| Effort expectancy | EE1 | 37(7.31) | 22(4.35) | 149(29.45) | 196(38.74) | 102(20.16) | 298(58.89) |
|  | EE2 | 39(7.71) | 76(15.02) | 148(29.25) | 166(32.81) | 77(15.22) | 243(48.02) |
|  | EE3 | 46(9.09) | 38(7.51) | 167(33.00) | 184(36.36) | 71(14.03) | 255(50.40) |
| Social influence | SI1 | 16(3.16) | 38(7.51) | 163(32.21) | 194(38.34) | 95(18.77) | 289(57.11) |
|  | SI2 | 48(9.49) | 28(5.53) | 123(24.31) | 182(35.97) | 125(24.70) | 307(60.67) |
|  | SI3 | 38(7.51) | 37(7.31) | 135(26.68) | 211(41.70) | 85(16.80) | 296(58.50) |
| Facilitating conditions | FC1 | 38(7.51) | 40(7.91) | 111(21.94) | 223(44.07) | 94(18.58) | 317(62.65) |
|  | FC2 | 15(2.96) | 36(7.11) | 131(25.89) | 228(45.06) | 96(18.97) | 324(64.03) |
|  | FC3 | 17(3.36) | 53(10.47) | 164(32.41) | 193(38.14) | 79(15.61) | 272(53.75) |
| Perceived risk | PR1 | 26(5.14) | 35(6.92) | 164(32.41) | 205(40.51) | 76(15.02) | 281(55.53) |
|  | PR2 | 33(6.52) | 15(2.96) | 172(33.99) | 177(34.98) | 109(21.54) | 286(56.52) |
|  | PR3 | 50(9.88) | 29(5.73) | 161(31.82) | 188(37.15) | 78(15.42) | 266(52.57) |
| Trust | TR1 | 21(4.15) | 27(5.34) | 164(32.41) | 188(37.15) | 106(20.95) | 294(58.10) |
|  | TR2 | 39(7.71) | 19(3.75) | 135(26.68) | 196(38.74) | 117(23.12) | 313(61.86) |
|  | TR3 | 30(5.93) | 19(3.75) | 115(22.73) | 255(50.40) | 87(17.19) | 342(67.59) |
| Behavioral intention | BI1 | 24(4.74) | 38(7.51) | 153(30.24) | 206(40.71) | 85(16.80) | 291(57.51) |
|  | BI2 | 11(2.17) | 29(5.73) | 130(25.69) | 223(44.07) | 113(22.33) | 336(66.40) |
|  | BI3 | 20(3.95) | 68(13.44) | 122(24.11) | 189(37.35) | 107(21.15) | 296(58.50) |

Abbreviations: n, number.

**Supplementary Table S5.** Univariate analysis of off-label drug acceptance among pregnant women with different characteristics (n = 506).

| **Characteristic** | **Category** | **behavioral intention score** | | **χ²** | ***P*** |
| --- | --- | --- | --- | --- | --- |
|  |  | **Median ≥4.0** | **Median <4.0** |  |  |
| Age (years) | 18-29 | 122 | 107 | 35.954 | **<0.001** |
|  | 30-39 | 127 | 73 |  |  |
|  | ≥40 | 18 | 59 |  |  |
| Monthly income (CNY) | <2000 | 38 | 44 | 3.728 | 0.292 |
|  | 2000-4000 | 103 | 83 |  |  |
|  | 4000-6000 | 73 | 55 |  |  |
|  | >6000 | 53 | 57 |  |  |
| Residence | Urban | 149 | 140 | 0.396 | 0.529 |
|  | Rural | 118 | 99 |  |  |
| Education level | Junior high school or below | 50 | 85 | 20.797 | **<0.001** |
|  | High school/technical secondary school | 37 | 28 |  |  |
|  | Junior college | 49 | 45 |  |  |
|  | Bachelor's degree or above | 131 | 81 |  |  |
| Employment status | Healthcare industry | 75 | 46 | 5.636 | 0.060 |
|  | Non-healthcare industry | 122 | 127 |  |  |
|  | Unemployed | 70 | 66 |  |  |
| Gestational trimester | <12 weeks | 73 | 71 | 11.840 | **0.003** |
|  | 12-28 weeks | 99 | 56 |  |  |
|  | >28 weeks | 95 | 112 |  |  |
| Parity | 0 | 102 | 86 | 1.118 | 0.773 |
|  | 1 | 107 | 96 |  |  |
|  | 2 | 36 | 31 |  |  |
|  | ≥3 | 22 | 26 |  |  |
| History of assisted reproduction | Yes | 28 | 15 | 2.876 | 0.090 |
|  | No | 239 | 224 |  |  |
| Hospital level for main antenatal care | Municipal or provincial general hospitals | 229 | 129 | 73.739 | **<0.001** |
|  | District/county-level hospital | 12 | 39 |  |  |
|  | Community/township health center | 5 | 18 |  |  |
|  | Maternal and child health hospital | 10 | 47 |  |  |
|  | Other | 11 | 6 |  |  |
| Drug history during pregnancy | Yes | 207 | 163 | 5.582 | **0.018** |
|  | No | 60 | 76 |  |  |
| Knowledge of off-label drug use before the research | Completely unaware | 58 | 61 | 2.138 | 0.343 |
|  | Heard of but unclear about the concept | 77 | 75 |  |  |
|  | Understood the definition | 132 | 103 |  |  |

Abbreviations: n: number, χ²: chi-square. Significant associations shown in **bold**.

**Supplementary Table S6.** Binary logistic regression sensitivity analysis of factors associated with high OLDU acceptance (n = 506).

| **Variable** | **B** | **SE** | **Wald** | ***P* value** | **OR** | **95% CI** |
| --- | --- | --- | --- | --- | --- | --- |
| UTAUT constructs (continuous) | | | | | | |
| Performance expectancy | −0.009 | 0.302 | 0.001 | 0.975 | 0.991 | 0.548 to 1.791 |
| Effort expectancy | 0.357 | 0.351 | 1.035 | 0.309 | 1.429 | 0.718 to 2.845 |
| Social influence | 0.017 | 0.400 | 0.002 | 0.965 | 1.017 | 0.464 to 2.230 |
| Facilitating conditions | 1.597 | 0.404 | 15.641 | **<0.001** | 4.938 | 2.238 to 10.896 |
| Perceived risk | 0.655 | 0.295 | 4.919 | **0.027** | 1.925 | 1.079 to 3.435 |
| Trust | 2.754 | 0.387 | 50.730 | **<0.001** | 15.700 | 7.359 to 33.496 |
| Gestational trimester (ref: <12 weeks) | | | | | | |
| 12-28 weeks | −0.505 | 0.474 | 1.134 | 0.287 | 0.604 | 0.238 to 1.528 |
| >28 weeks | −0.861 | 0.438 | 3.863 | **0.049** | 0.423 | 0.179 to 0.998 |
| Hospital level (ref: Municipal/provincial general hospitals) | | | | | | |
| District/county-level hospital | −1.128 | 0.588 | 3.683 | 0.055 | 0.324 | 0.102 to 1.024 |
| Community/township health center | 0.777 | 0.785 | 0.981 | 0.322 | 2.175 | 0.467 to 10.130 |
| Maternal and child health hospital | −1.156 | 0.653 | 3.131 | 0.077 | 0.315 | 0.087 to 1.132 |
| Other | 1.869 | 1.117 | 2.799 | 0.094 | 6.479 | 0.726 to 57.832 |
| Medication history during pregnancy (ref: Yes) | | | | | | |
| No | −0.389 | 0.397 | 0.956 | 0.328 | 0.678 | 0.311 to 1.477 |
| Age (ref: 18-29 years) | | | | | | |
| 30-39 years | 0.341 | 0.390 | 0.763 | 0.382 | 1.406 | 0.655 to 3.020 |
| ≥40 years | −0.216 | 0.584 | 0.137 | 0.711 | 0.806 | 0.257 to 2.530 |
| Education level (ref: Junior high school or below) | | | | | | |
| High school/technical secondary school | 0.472 | 0.600 | 0.619 | 0.432 | 1.603 | 0.495 to 5.195 |
| Junior college | 0.127 | 0.547 | 0.054 | 0.817 | 1.135 | 0.389 to 3.314 |
| Bachelor's degree or above | 0.580 | 0.486 | 1.427 | 0.232 | 1.786 | 0.690 to 4.626 |

Abbreviations: B, unstandardized regression coefficient;OR, odds ratio; CI, confidence interval; BI, behavioral intention; OLDU, off-label drug use. Significant associations shown in **bold**.
